# Developing Asthma-Friendly Childcare Centers with Online Training and Evaluation

**DOI:** 10.3389/fpubh.2016.00039

**Published:** 2016-03-16

**Authors:** Alexandra Catherine Hayes Nowakowski, Henry Joseph Carretta, Nicole Pineda, Julie Kurlfink Dudley, Jamie R. Forrest

**Affiliations:** ^1^Behavioral Sciences and Social Medicine, Florida State University College of Medicine, Tallahassee, FL, USA; ^2^Florida Department of Health, Tallahassee, FL, USA

**Keywords:** asthma, childcare, online training, program evaluation, Florida

## Abstract

In 2011, the Florida Asthma Coalition (FAC) began offering its Asthma-Friendly Childcare Center (AFCC) training online. This course teaches childcare center employees the fundamentals of effective asthma management. It covers basic asthma physiology, ways to recognize asthma attacks, techniques to help children experiencing attacks, and strategies to create healthy environments for asthmatics. A team of health services researchers evaluated both years of the online training. Evaluators used a quasi-experimental design with pretest, posttest, and follow-up assessment. Questions measured knowledge gain and retention, user satisfaction, and implementation of management strategies. Over 650 people from nearly all 67 Florida counties took AFCC training online between 2011 and 2013. Test scores improved by a minimum of 11% points in all program years evaluated. Gains in both knowledge and confidence were substantial and highly significant across years. While individual trainees did forget some content on follow-up, they seemed to retain the specific messages most relevant for their own workplaces. Most trainees also planned to implement multiple management strategies recommended by the training. A large majority of participants rated the training as excellent on all quality metrics, including relevance of content and time efficiency of the online format. Nearly all respondents perceived the training as useful for both providing improved care and fulfilling licensure or certification requirements. Many participants also indicated that their centers would pursue formal certification as AFCCs *via* the program offered by FAC. The online AFCC course performed strongly in its first years, yielding both high participant satisfaction and substantial improvement in workplace asthma management activity. This training holds promise for introducing and improving multidimensional asthma management strategies at childcare facilities nationwide.

## Introduction

In 2011, the Florida Asthma Coalition (FAC) began offering its Asthma-Friendly Childcare Center (AFCC) training online. This single-session course was developed in partnership with St. Petersburg College, which also served as the data steward for pilot sessions of the training. The 1-h training covers basic concepts of asthma physiology, ways to recognize that a child is having an asthma attack, techniques to help a child who is having an asthma attack, and strategies to help create healthier environments for children with asthma.

Participants in the course take a pretest to capture their knowledge at baseline. After completing the course, participants take a posttest in which they respond to the same knowledge questions, as well as a supplemental survey with questions about the quality of the course itself. Starting in spring 2012, participants have also been sent a follow-up survey roughly 2 months following completion of the course. This follow-up survey tracks knowledge retention from taking the course, as well as changes in asthma management behavior implemented by course participants.

The AFCC program has been active from summer 2011 through the present. This article focuses on results from the preliminary evaluation of AFCC. Initial evaluation efforts used data from the first two program years: 2011–2012 and 2012–2013. A subsequent evaluation was conducted with the same instruments for the online version of the training using a modified content management platform in the 2013–2014 program year.

### Background

The prevalence of childhood asthma in the United States has increased steadily over time (1). Asthma morbidity and mortality rates have amplified along with prevalence, especially in underserved communities in both urban and rural areas due to lack of resources (1). Studies suggest that management of childhood asthma with a multidisciplinary population and clinical approach is a successful method to prevent lifelong detrimental effects (2). Successful intervention strategies include asthma education, parenting workshops, therapy management, environmental trigger mitigation, health-care quality improvement, and direct communication with physicians (2).

Zahran et al. (3) stress that educational efforts are more likely to succeed when multiple management strategies and service populations are targeted. In addition to reducing financial barriers and other access constraints and helping people with asthma use accessible resources in an evidence-based manner, the most successful asthma management programs also target the knowledge and behaviors of other people in patients’ lives (3). For young children, these individuals are likely to include immediate family members, health-care providers, and day care employees.

Educational interventions with families were some of the earliest to demonstrate success. Multiple teams have developed asthma self-management curricula for urban low-income families, both in general (4) and specifically for Latino and African-American families (5). These interventions have shown success improving knowledge, preventing symptoms, and making it possible for children to be physically active, as well as decreasing utilization of emergency health-care services. Both interventions engaged family members as well as children themselves, suggesting that educating professionals who work with young children may help to increase the effectiveness of asthma management outside the home. Some programs have also achieved success by engaging family members and care providers simultaneously. Garbutt et al. (6) assessed a program in which a certified asthma educator trained parents *via* telephone. The 6-month training course yielded significant positive changes in asthma management competencies and behaviors.

Several research teams have assessed the effectiveness and promise of computer-based training for asthma management. Early efforts to use computer technology to help children develop good self-management skills yielded positive results (7). Participants in the technology intervention demonstrated improved knowledge of asthma, more consistent self-management behaviors, and lower utilization of emergency services (7). These early trainings were taken at desktop computing stations and were not accessible in an online format. Other teams have since built on this work with a variety of multimedia trainings, often focusing on underserved populations in urban areas and the providers who care for them. Homer et al. (8) worked with a hospital-based primary care clinic and affiliated neighborhood health center to implement and evaluate a multimedia program for asthma management. Their training significantly improved knowledge for both children and their parents, but not symptom severity or health-care utilization. By contrast, Shegog et al. (9) found that their computer-assisted program did not significantly improve knowledge compared to other programs. However, their program proved significantly more helpful in building positive behaviors for self-regulation, prevention, and treatment. Participants found both trainings motivating and rewarding (8, 9). Positive results across multiple domains were achieved by McPherson et al. (10), who worked only with children and incorporated a higher level of detail in the information presented with their computer-based program. Children’s knowledge, behavior, and confidence all improved significantly with this training.

Building on the increasing level of success observed with newer versions of traditional multimedia trainings using curricula on CD-ROM, more recent efforts have extended the successes of these trainings to online and hybridized formats. Early data suggest a possible advantage for online training formats that is likely to persist due to progressively higher levels of connectivity in younger populations. Web-based education has yielded excellent results in domains such as consistent use of controller medications, reduced use of reliever medications, and fewer urgent physician visits (11). Cicutto et al. (12) evaluated a hybrid intervention program that included in-person, computerized, and web-based training for primary care clinicians. This intervention offered clinical support tools, patient education materials, and spirometry assessment demonstrations. A self-reported assessment showed significant improvement in confidence levels of primary care providers and team members offering better quality care for asthma.

Although health-care providers were engaged in some of these multimedia trainings, none of these efforts directly engaged childcare workers or incorporated a systems perspective. However, Pronovost et al. (13) evaluated a highly successful comprehensive asthma care and patient safety program that used a systems approach to health promotion. The research team concluded that high patient health-care quality is achieved when organizational management closely monitors care processes and safety by *via* mutually supportive committees for quality, safety, and service. Araújo-Martins et al. (14) reviewed environmental and psychological influences on respiratory health by conducting research in two phases and proposing a new airway ventilation method. The two-phase International Study of Asthma and Allergies in Children (ISAAC) study offered insight into asthma management factors across multiple environmental and behavioral domains relevant for air quality in microenvironments where children spend time and recommended intervention strategies for addressing these in systems context.

Reviewing extant literature supports four key elements for new interventions. First, these studies suggest that a multidisciplinary approach offers important advantages for the management of asthma. Second, they recommend a specific focus on training professionals who work with children both within and outside of the home. Third, they indicate multiple advantages in the use of multimedia and web-based interventions. Fourth, they demonstrate the benefits of incorporating a focus on broad governance initiatives targeting environmental management at the organizational level.

The AFCC training developed by the Florida Asthma Program (FAP) in collaboration with St. Petersburg College incorporates these four principles in its integrative approach to asthma management. To date, the AFCC training is the first of its kind – an online intervention that not only assesses but also builds capacity for asthma management in childcare centers. However, an instrument for evaluating readiness to work with young children with asthma in day care settings has recently been released.

The Preparing for Asthma in Child Care (PACC) questionnaire uses national guidelines and standards along with evidence from scientific literature, onsite case studies, and key informant interviews to assess how prepared childcare centers are to work with young asthmatics (15). The PACC asks about 43 different readiness elements across seven key domains of asthma management: smoking exposure, medical consultation, trigger management, medication access, care planning, employee training, and physical activity (15). Evidence from evaluation of the PACC suggests that trainings, such as AFCC, can substantially help to improve asthma management processes and outcomes in childcare environments (15).

## Materials and Methods

The overall goal of the evaluation was to see how well the online version of the AFCC training builds capacity to create asthma-friendly environments for young children in day care settings. Evaluation of the AFCC training addressed the following specific questions:

1. How many people are participating in the AFCC training?
2. How much knowledge are participants gaining from the AFCC training?
3. How satisfied are participants with the AFCC training?
4. How much have participants changed their behavior/taken action since participating in the AFCC training?

The FAC will share evaluation results with key stakeholders including training participants, program partners, FAC Childcare Workgroup members, local asthma coalitions, and FAP staff at the Centers for Disease Control and Prevention (CDC). Findings will be used to improve course content and evaluation tools in future years.

Scoring reports for pretest and posttest data were shared with evaluators by FAP staff. These scoring reports consisted of Excel spreadsheets with one row for each participant. Pretest and posttest responses were recorded by question, and demographic information about participants was also captured. Information about asthma management in each content domain was captured using matrix questions with Likert response scales, indicating increasing levels of agreement (e.g., “1 – not at all helpful” to “5 – extremely helpful”). These data were recoded in Excel to reflect correct and incorrect responses (for knowledge questions) or level of agreement (for quality questions). Follow-up survey data were downloaded from SurveyMonkey into Excel, where they were likewise recoded into dummy or polytomous categorical variables as appropriate. Data on perceived course quality were analyzed by computing frequencies of spreadsheet cell values. Knowledge gain from pretest to posttest was assessed using paired *T*-tests to check for significant differences in scores for each pretest and posttest question, as well as overall scores. Likewise, paired *T*-tests were conducted to assess knowledge gain or loss between posttest and follow-up.

## Results

A total of 296 people took the online AFCC training between September 1, 2011 and June 30, 2012; pretest and posttest data are available for all 296 of these participants. Of those individuals, 96 also completed the 2-month follow-up survey. Only people who participated through June 30 were included in the 2012 analysis due to timing constraints for outcomes reporting during Grant Year 3.

A total of 365 people took the online AFCC training between September 1, 2012 and August 31, 2013. Of these individuals, 94 reported working for Head Start Programs. Pretest and posttest data are available for all 365 of these participants. Of the 365, 113 also completed the 2-month follow-up survey, which concluded in early November 2013 for the August cohort. Participants were distributed across multiple Florida counties, as shown in Table 1. A majority of respondents were employed in Alachua, Bay, Broward, Charlotte, Duval, Hillsborough, Miami-Dade, Orange, and Pinellas counties.

**Table 1:** **Distribution of AFCC participants by county**.

County	Participants (2011–2012)	Participants (2012–2013)
Alachua	18	3
Baker	7	0
Bay	1	0
Brevard	5	4
Broward	18	22
Charlotte	3	1
Citrus	1	0
Clay	5	6
Collier	0	9
Columbia	3	0
DeSoto	1	0
Duval	25	1
Escambia	7	2
Hardee	3	1
Hernando	0	1
Hillsborough	19	25
Indian River	1	0
Jackson	2	0
Lafayette	0	1
Lake	6	0
Lee	9	152
Leon	9	1
Marion	11	0
Martin	3	0
Miami-Dade	26	3
Nassau	3	0
Okaloosa	2	15
Orange	16	75
Osceola	3	8
Palm Beach	4	3
Pasco	7	1
Pinellas	18	3
Polk	4	4
Santa Rosa	2	9
Sarasota	2	1
Seminole	12	9
St. Johns	1	0
St. Lucie	2	1
Taylor	0	1
Volusia	12	1
Wakulla	0	1
Walton	0	1
Unreported	24	0
Total	296	365

Responses from knowledge questions on the pretest, posttest, and follow-up survey were compared to assess knowledge gain and retention. These data were compared using paired *T*-tests to detect significant differences in average percentage of respondents answering each question correctly between pretest and posttest, as well as between posttest and follow-up for knowledge questions that were repeated on the follow-up survey. Results from statistical testing are displayed in Table 2. Item-specific scores are expressed as the percentage of trainees answering each question correctly. Total scores are expressed as the average percentage of questions answered correctly by all trainees. Differences in scores are expressed in percentage points for both item-specific and total metrics.

**Table 2:** **Observed changes in knowledge, pretest to posttest**.

Question	Difference 2011–2012 (*n* = 296)	Difference 2012–2013 (*n* = 365)	Interpretation
What part of the body is affected by asthma?	+1.68%*	+1.36%^†^	Significant improvement in both years
Select the two main types of asthma medications	+2.13%***	+11.2%***	Significant improvement in both years
Which type should children with asthma always have on hand at your facility?	+18.6%***	+17.0%***	Significant improvement in both years
Name the first thing you should do if a child shows asthma symptoms	+4.39%***	+5.75%***	Significant improvement in both years
Name a sign that the child’s asthma is getting worse and 911 should be called	+2.70%*	+1.92%*	Significant improvement in both years
What is an asthma action plan?	+1.82%***	+1.42%***	Significant improvement in both years
Which of these things can trigger an asthma attack if present in your facility?	+4.12%***	+3.53%***	Significant improvement in both years
Identify a way to manage children with asthma in your childcare facility	+3.04%*	−0.274%	No consistent change across years
Which of the following triggers can cause an asthma episode?	+0.337%	+1.92%*	No consistent change across years
During an asthma episode, what happens to the airways?	+7.09%***	+7.40%***	Significant improvement in both years
Which of these is not a way to control asthma triggers in a childcare facility?	+11.1%***	+10.4%***	Significant improvement in both years
What does the term “spacer” describe?	+2.74%***	+16.4%***	Significant improvement in both years
Total knowledge score	+12.8%***	+11.0%***	Significant improvement in both years

*^†^*p* < 0.10*.

*p < 0.05.

**p < 0.01.

***p < 0.001.

In both years, trainees showed significant knowledge gain from pretest to posttest on all questions except two. No consistent change was observed across program years for knowledge of management strategies or triggers. In addition, participants’ total scores increased markedly from pretest to posttest, with a minimum of 11% improvement in both program years. In 2011–2012, average posttest scores increased to 95% from a baseline average score of 82% on pretest. In 2012–2013, similar increases were observed, from an average baseline of 84% on pretest to an average posttest score of 95%.

Results were also compared for knowledge questions that matched on the posttest and follow-up survey. Follow-up assessment of knowledge yielded poor outcomes, with trainees showing slightly decreased knowledge on two out of three questions. Given the smaller battery of questions (3 versus 11) as well as the smaller sample of people (96 versus 296 in 2011–2012 and 113 versus 365 in 2012–2013) who completed the online follow-up survey compared to the pre- and posttests, caution should be exercised in interpreting these results.

Trainees were asked to indicate their level of confidence about specific asthma management activities on the follow-up survey. Differences in scores are expressed as points in a five-point attitudinal scale. Table 3 displays results from these analyses. The average baseline score for item-specific confidence questions in 2011–2012 was 4.01 on pretest and increased to an average item-specific score of 4.53 on posttest. Changes in scores were similar in 2012–2013, with an average baseline item-specific confidence score of 3.91 on pretest that increased to an average item-specific score of 4.40 on posttest. All confidence scores are out of 5 possible points, with 5 points indicating the highest level of confidence and 1 point indicating the lowest level.

**Table 3:** **Observed changes in beliefs, pretest to posttest**.

Question	Difference 2011–2012 (*n* = 296)	Difference 2012–2013 (*n* = 365)	Interpretation
I believed asthma is a serious medical condition that could be controlled	+0.198**	+0.237***	Significant improvement in both years
I felt it was my role or responsibility to help a child with asthma	+0.259***	+0.312***	Significant improvement in both years
I felt comfortable administering medication to a child with asthma symptoms	+0.593***	+0.559***	Significant improvement in both years
I felt confident in my efforts to manage asthma triggers in my facility	+0.716***	+0.645***	Significant improvement in both years
I felt prepared to help a child during an asthma episode	+0.716***	+0.699***	Significant improvement in both years
I believed talking with parents could prevent an episode from getting worse	+0.259***	+0.355***	Significant improvement in both years
I felt comfortable assisting a child with asthma	+0.704***	+0.602***	Significant improvement in both years

**p < 0.01.

***p < 0.001.

Gains in confidence about asthma management activities after taking the training were highly significant across all seven questions in both program years. Taken together with findings from knowledge retention questions, these results suggest that while individual trainees may not remember every specific piece of information they learned in the training, they are likely retaining the specific training messages that are most relevant for implementation in their own work settings.

Participants were asked to rate the quality of specific elements of the training. Distribution of ratings across quality categories is shown in Table 4. In both program years, a large majority of participants found the training excellent across the board. Most other respondents rated elements of the training uniformly as very good. No more than one participant in either program year rated the training as poor, and no more than one rated any item as fair.

**Table 4:** **Participant perceptions of AFCC training quality**.

Question	Very good or excellent 2011–2012 (*n* = 96)	Very good or excellent 2012–2013 (*n* = 113)
Fulfilled identified goals and objectives	280 (100%)	331 (97%)
Provided information at appropriate level	263 (94%)	330 (97%)
Provided relevant knowledge for career	266 (95%)	329 (96%)
Provided environment conducive to learning	259 (92%)	327 (96%)
Provided high-quality learning experience	260 (93%)	328 (96%)
Provided accessible location	265 (94%)	327 (96%)

On the posttest, trainees were asked to indicate how useful the training was for their professional development and activities. Nearly all respondents indicated that the training was useful for both providing improved care and fulfilling licensure or certification requirements. The 2-month follow-up survey also asked questions about the utility of the training; these questions were mapped to specific asthma management activities. In both program years, a large majority of respondents found the training either extremely influential or very influential in helping them build asthma management skills.

The 2-month follow-up survey asked participants to indicate whether or not they had implemented specific asthma management strategies since taking the training. Although we had fewer people taking the follow-up survey than completing the course in all evaluated years, we did not observe significant differences in demographics between people who took the follow-up assessment and people who did not. Results are shown in Table 5. Responses varied somewhat within and across program years. Across program years, asthma leadership teams, using communications forms with parents, completing trigger assessment checklists, establishing trigger assessment schedules, and passing asthma management policies were the most consistently popular interventions.

**Table 5:** **Reported implementation of asthma management behaviors**.

Strategy	Yes or already in place 2011–2012 (*n* = 96)	Yes or already in place 2012–2013 (*n* = 113)
Have an asthma leadership team	25 (32%)	44 (52%)
Request asthma action plans	62 (81%)	15 (18%)
Keep asthma action plans on file	55 (71%)	14 (17%)
Use communications forms with parents	34 (44%)	42 (50%)
Display educational posters	26 (34%)	35 (42%)
Provide literature/resources to parents	38 (49%)	27 (32%)
Refer parents to community services	45 (58%)	23 (27%)
Receive and heed air quality alerts	25 (32%)	49 (58%)
Completed trigger assessment checklist	34 (44%)	35 (42%)
Established trigger assessment schedule	39 (51%)	42 (50%)
Passed asthma management policy	25 (32%)	37 (44%)

Smaller numbers of trainees indicated that these activities were already in place at their facilities before they took the training. Likewise, several participants were not sure of which activities were already in place or currently being implemented. In 2011–2012, about the same amount of trainees answered “yes” and “no” for each question. The following program year, however, “yes” became the most common response to each question.

Finally, trainees were asked about their use of the guidance resources provided by the FAC, as well as their intent to pursue the AFCC certification for their centers. While many participants were already using the resource guide, fewer were certain of their center’s intent to pursue AFCC recognition. However, a great many more were not sure whether or not their center would pursue the designation. Comparatively few respondents said that their center had no intention of pursuing the AFCC recognition opportunity. In 2012–2013, five respondents also said that they had already secured AFCC recognition for their centers prior to taking the training.

## Discussion

Overall, findings from this evaluation suggest that the online AFCC training offers valuable information in an accessible format that makes learning easy. The quality of the training may explain why it attracted substantial numbers of people from almost all of Florida’s 67 counties in its first 2 years of availability. Likewise, the diversity of professions represented among people who took the course suggests that it is useful not just for people who work in traditional childcare centers but also for anyone else who interacts with children affected by asthma.

Preliminary results for knowledge retention from posttest to follow-up are weak. However, the small number of follow-up questions may not capture the types of information from the course that have actually proven most useful to trainees. This is especially likely since the specific questions included in the follow-up to measure knowledge address behaviors and strategies rather than asthma pathology or possible triggers. Likewise, results from questions about which management strategies trainees have implemented suggest that knowledge retention and the ability to translate this knowledge into action may be stronger than indicated by the follow-up questions on asthma basics.

Given the strength of results on the posttest, we were unsurprised to see a small slip in scores on follow-up due to the time lag. However, we also note that assessment of knowledge change from posttest to follow-up was not as thorough as assessment of knowledge gain from taking the course, due to the much smaller sample size and smaller number of questions. We might have seen overall results more similar to those obtained on posttest if we had repeated the full battery of knowledge questions instead of choosing three. In either case, participants retained a high level of knowledge on follow-up despite not scoring quite as highly as they did on posttest.

Participants corroborated the quality of the training almost unanimously, rating it highly on a variety of different quality assessments and consistently stating that it enhanced their professional lives. Likewise, trainees gave overwhelmingly positive reports of how their confidence about asthma management changed after taking the course. These increases in confidence are solidly borne out by available data on behavior change – despite the comparatively small sample sizes of only 96 and 113 people for the follow-up surveys, implementation of recommended management strategies appeared to be widespread. No behavior change was reported by fewer than 17% of participants. More typical rates of behavior change were well above 50%, with a maximum of 82%.

Increases in participant’s knowledge from pretest to posttest also suggest that the training is highly effective at developing multidimensional competencies in asthma management. In both program years, all but two of the knowledge gain questions showed highly significant improvement in scores upon posttest, with *p*-values below 0.01 and often close to 0. Likewise, data collected on time required to complete the course suggest that the online AFCC training is an efficient means of delivering this content and provides busy professionals with the flexibility required to fit continuing education activities into their work schedules.

Many people who have taken the course have reviewed the supplementary materials (e.g., Asthma-Friendly Childcare Resource Guide) and are using them at work. A substantial number of participants also indicated that their centers had already decided to pursue the AFCC recognition, and almost twice as many indicated that pursuing the recognition was a possibility by choosing “not sure.” The high number of “not sure” responses on this question may owe in part to phrasing – some respondents may not have decision-making authority about whether or not their centers pursue the certification, despite independent interest in taking advantage of this opportunity.

Overall, data quality was a leading strength of the AFCC training and its component evaluation activities. The high level of data quality achieved across multiple years of AFCC implementation appeared to stem largely from the online delivery system developed by St. Petersburg College and linkage with SurveyMonkey follow-up assessment managed by FAP. Completeness of data was also a substantial strength of the training and our evaluation thereof for pretest and posttest, and a modest strength for follow-up assessment as well. Given adequate sample sizes for records with three timepoints’ worth of data, we could make reasonable inferences about the long-term potential for positive impact on asthma management behaviors and policies without imputing any values for participants who were missing data on the follow-up assessment. However, we do note that our projections about long-term impact could be much stronger given more timepoints of follow-up data collection.

## Conclusion

The AFCC training performed extremely well in its first years and holds tremendous promise for changing the landscape of asthma management at childcare facilities nationwide. To strengthen the training further, evaluators offered recommendations based on results from preliminary evaluation. Researchers suggested four action items for FL DOH program staff and St. Petersburg College partners. Recommendations are outlined below, along with implementation strategies for each.

### Revisit the Issue of Knowledge Loss on Certain Items between Posttest and Follow-up with Data from Future Program Years

The slight knowledge loss observed in this evaluation did not present a major concern due to unmatched sample sizes between posttest and follow-up. Evaluators projected that analyzing complete data for all cohorts in future program years would afford better understanding of why participants may lose some knowledge over time. However, evaluators also noted that some score attrition might continue to occur as a function of regression to the mean, given extremely high scores observed on posttesting for the preliminary evaluation.

### Investigate Why Many Participants Are “Not Sure” Whether or Not Their Centers Will Pursue the AFCC Recognition Opportunity

Wording of the original evaluation question made causal inference impossible regarding why people felt unsure, because it only assessed organizational strategy as opposed to participant interest. Evaluators and FL DOH program staff changed this question language slightly to reflect participants’ actual interest in the certification.

### Continue to Monitor Which Behavior Changes Participants Are Implementing at Lower Rates

Strong results for behavior change across the board did not indicate taking in-depth action to address discrepancies between rates of behavior change on specific measures. However, analysis of additional cohorts in future program years may reveal systemic trends in which behavior changes might not be sufficiently emphasized in the training or easily achievable in specific workplaces. The latter point may become especially salient if AFCC is offered in other states.

### Market the Online Training as Aggressively as Resources Permit

Evaluation results from the online course’s first year suggested that AFCC offers an efficient, effective, engaging, impactful, and empowering opportunity for childcare professionals to increase their skill in managing children’s asthma. Given the ease of offering the training and the quality of evaluation data the online delivery method provides, evaluators recommended making every effort to increase exposure and enrollment for this training in years to come.

Enrollment data for more recent program years show a consistent upward trend in the popularity of AFCC. Program managers and evaluators have also worked actively to disseminate information about the AFCC training to public health professionals from other states. Successes and lessons learned from the program are shared regularly at national conferences, with chronic disease prevention and management coalitions, and *via* online public health listservs. As AFCCs reach continues to expand in Florida, other states may wish to explore the utility of adapting this training for use in their own childcare facilities.

## Author Contributions

AN completed all data management and analysis, wrote the first drafts of most manuscript sections, and edited the whole manuscript. HC assisted with all data management and analysis and contributed edits during the manuscript writing process. NP wrote the literature review and contributed edits during the manuscript writing process. JD designed the intervention, supplied data for analysis, and contributed edits during the manuscript writing process. JF assisted with designing the intervention, supplied data for analysis, and contributed edits during the manuscript writing process.

## Conflict of Interest Statement

The authors declare that the research was conducted in the absence of any commercial or financial relationships that could be construed as a potential conflict of interest.
